# Multi-atlas segmentation and quantification of muscle, bone and subcutaneous adipose tissue in the lower leg using peripheral quantitative computed tomography

**DOI:** 10.3389/fphys.2022.951368

**Published:** 2022-10-14

**Authors:** Sokratis Makrogiannis, Azubuike Okorie, Angelo Di Iorio, Stefania Bandinelli, Luigi Ferrucci

**Affiliations:** ^1^ Math Imaging and Visual Computing Lab, Division of Physics, Engineering, Mathematics and Computer Science, Delaware State University, Dover, DE, United States; ^2^ Antalgic Mini-invasive and Rehab-Outpatients Unit, Department of Innovative Technologies in Medicine & Dentistry, University “G.d’Annunzio”, Chieti-Pescara, Italy; ^3^ Geriatric Unit, Local Health Unit Tuscany Centre, Florence, Italy; ^4^ National Institute on Aging, National Institutes of Health, Baltimore, MD, United States

**Keywords:** tissue segmentation, tissue quantification, multi-atlas techniques, subject movement, clinical application, pQCT

## Abstract

Accurate and reproducible tissue identification is essential for understanding structural and functional changes that may occur naturally with aging, or because of a chronic disease, or in response to intervention therapies. Peripheral quantitative computed tomography (pQCT) is regularly employed for body composition studies, especially for the structural and material properties of the bone. Furthermore, pQCT acquisition requires low radiation dose and the scanner is compact and portable. However, pQCT scans have limited spatial resolution and moderate SNR. pQCT image quality is frequently degraded by involuntary subject movement during image acquisition. These limitations may often compromise the accuracy of tissue quantification, and emphasize the need for automated and robust quantification methods. We propose a tissue identification and quantification methodology that addresses image quality limitations and artifacts, with increased interest in subject movement. We introduce a multi-atlas image segmentation (MAIS) framework for semantic segmentation of hard and soft tissues in pQCT scans at multiple levels of the lower leg. We describe the stages of statistical atlas generation, deformable registration and multi-tissue classifier fusion. We evaluated the performance of our methodology using multiple deformable registration approaches against reference tissue masks. We also evaluated the performance of conventional model-based segmentation against the same reference data to facilitate comparisons. We studied the effect of subject movement on tissue segmentation quality. We also applied the top performing method to a larger out-of-sample dataset and report the quantification results. The results show that multi-atlas image segmentation with diffeomorphic deformation and probabilistic label fusion produces very good quality over all tissues, even for scans with significant quality degradation. The application of our technique to the larger dataset reveals trends of age-related body composition changes that are consistent with the literature. Because of its robustness to subject motion artifacts, our MAIS methodology enables analysis of larger number of scans than conventional state-of-the-art methods. Automated analysis of both soft and hard tissues in pQCT is another contribution of this work.

## 1 Introduction

Accurate segmentation of tissues using medical imaging is key for the quantification of changes in the structure and composition of tissues, which may result from diseases, aging, and other risk factors related to the tissue(s) in question (Cordova et al., 2014; Töpfer et al., 2015; Owen et al., 2019; Rodrigues and Pinheiro 2019; Wong and Manske 2020). Recent advances of artificial intelligence (AI) in the field of medical imaging have also drawn the interest of researchers in the application of computer techniques in the area of bone and muscle imaging (Burns et al., 2020). In clinical studies, segmentation of bone, muscle, and adipose tissue can be used for computing objective measures and descriptors of body composition and for exploring the causes and effects of differences of these descriptors between subject groups (Lauretani et al., 2008; Makrogiannis et al., 2018).

In the past 2 decades, peripheral quantitative computed tomography (pQCT) and high resolution peripheral quantitative computed tomography (HR-pQCT) have emerged as essential technologies for segmentation and quantification of bone, muscle and adipose tissue properties at the diaphyseal regions of the limbs. Segmentation of hard and soft tissues in pQCT and HR-pQCT imaging has been used to assess the effects of type-2 diabetes mellitus (T2DM) (Starr et al., 2018), osteoporosis (Simon et al., 2022) and osteoarthritis (Chen et al., 2018), to establish measures for characterizing sex-, ethnic-, site-, and age-related outcomes (Gabel et al., 2018), to study the effect of exercise on the muscle and fat cross-sectional areas (Rowe et al., 2019), and in studies of aging and age-related diseases (Chow et al., 2022; Liu et al., 2022). A challenge in pQCT-based segmentation is subject movement and the associated motion artifacts. Subject movement, subtle or obvious, occurs frequently in standard pQCT and HR-pQCT scans (Wong 2016). It may degrade the image quality and affect the assessment of bone and muscle properties (Pialat et al., 2012; Chan et al., 2018). pQCT motion has been assessed by visual inspection followed by a pass or fail decision. Usual criteria are the presence of discontinuities and streaks at the cortical bone and changes in intensity of trabecular bone. Motion streaks originating from the cortical bone extend into the muscle. Quantitative evaluation methods have been proposed for pQCT (Blew et al., 2014) and HR-pQCT (Pauchard et al., 2011; Sode et al., 2011). Thresholding (Blew et al., 2014) and watershed segmentation techniques (Wong 2016) have been employed to identify and assess motion streaks in the muscle. Motion artifact correction and automatic segmentation are desirable.

**FIGURE 1:**
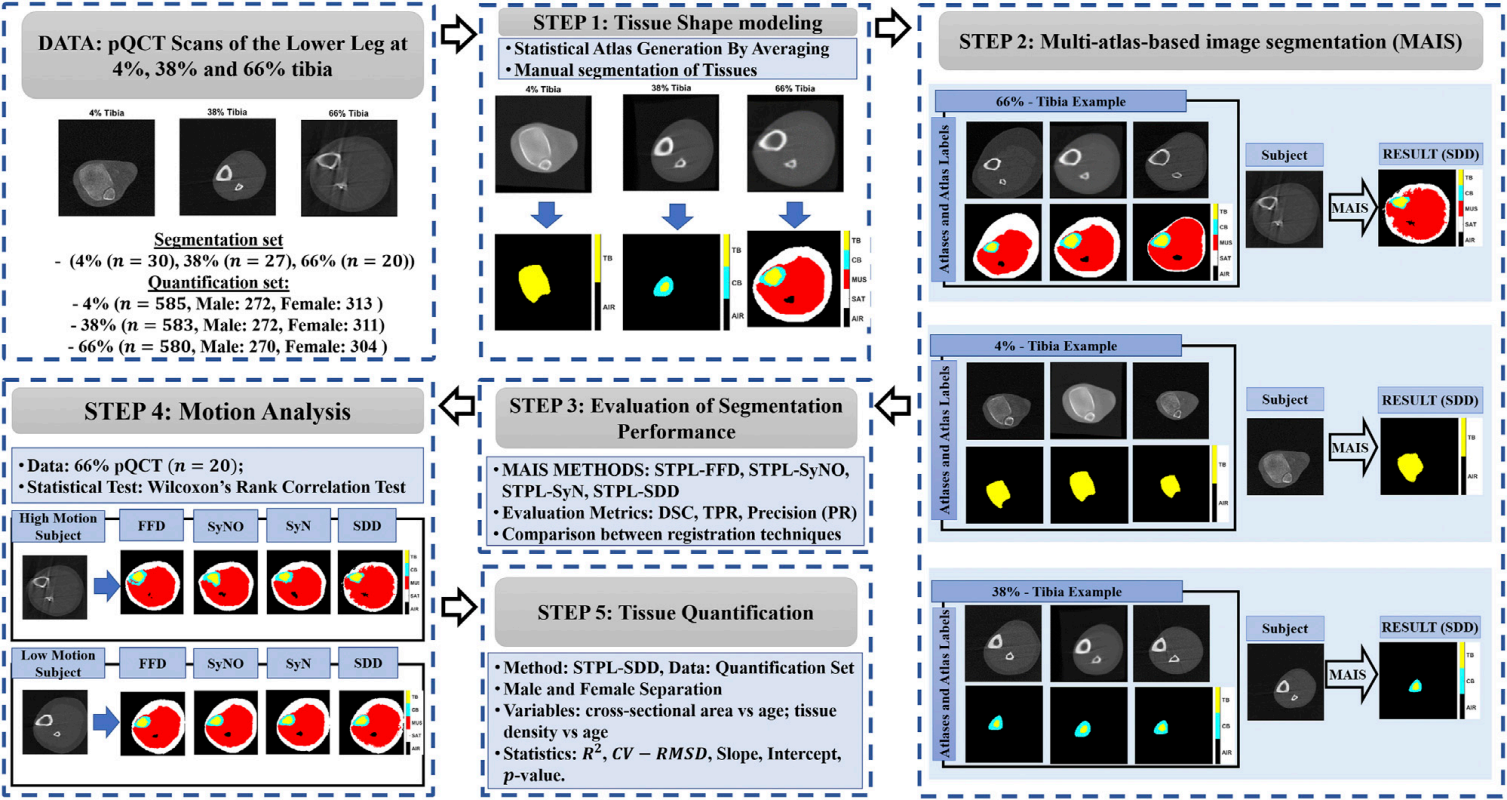
Main components of the proposed framework. STAPLE (STPL), free-form deformation (FFD), symmetric diffeomorphic demons (SDD), symmetric normalization (SyN), symmetric normalization - only (SyNO), dice similarity coefficient (DSC), true positive rate (TPR), precision (PR) squared-Spearman’s correlation coefficient (*R* ^2^), and coefficient of variation - root mean squared difference (CV-RMSD).

However, to the best of our knowledge, there is no previous report in the literature on pQCT segmentation techniques explicitly addressing subject movement and the limited contrast-to-noise ratio that are characteristic of this modality. In this work we propose to address this gap by developing a multi-atlas image segmentation (MAIS) framework (Rohlfing et al., 2001, 2005; Shen and Hammer, 2002; Langerak et al., 2010; Sotiras et al., 2013; Iglesias and Sabuncu 2015) for identification of soft and hard tissues in pQCT scans of the lower leg. The MAIS framework includes the stages of statistical atlas generation, linear and non-linear registration, and label fusion for tissue segmentation. In these stages we use pQCT images of the lower leg at 4%, 38%, and 66% of the tibial length. We validated segmentation performance against manual reference masks using the Dice Similarity Coefficient (DSC). We evaluated the performance of multiple atlas-based tissue segmentation techniques and an established model-based tissue segmentation technique. We expect that the segmentation performance of multi-atlas based methods is largely unaffected by motion. We have also applied our framework to a larger out-of-sample dataset and reported our results on age-related tissue composition changes. Figure 1 summarizes the main components of the proposed framework and related experiments.

## 2 Our methodology

### 2.1 Atlas-based tissue segmentation

**FIGURE 2:**
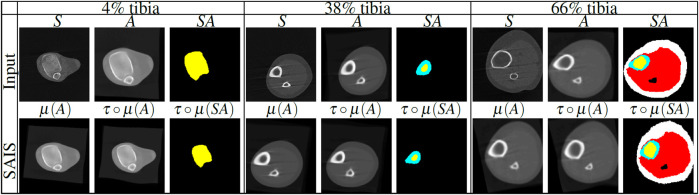
Illustration of single atlas based image segmentation (SAIS) stages of lower leg scans at 4%, 38% and 66% tibia. First row: subject (*S*), statistical atlas (*A*) and segmented atlas label map (*SA*) for each tibial length that are used as inputs for segmentation. Second row: linear registration, nonlinear registration and label propagation output. The tissue labels are color-coded as follows: trabecular bone (yellow), cortical bone (cyan), muscle (red), and SAT (white). *μ* denotes linear transformation, *τ*◦*μ* denotes the composition of linear (*μ*) with nonlinear (*τ*) transformations.

We formulate the problem of atlas-based segmentation next. Given a subject *S*, an atlas *A* and its atlas label map *SA*, we aim to produce the segmentation of *S* by warping the atlas to the spatial domain of the subject. This stage is called image registration, and is followed by pixel-wise assignment of tissue labels from the warped segmentation atlas to the subject (Figure 2). Since a single atlas is used for segmentation, we refer to this method as *single atlas image segmentation (SAIS)*.

**FIGURE 3:**
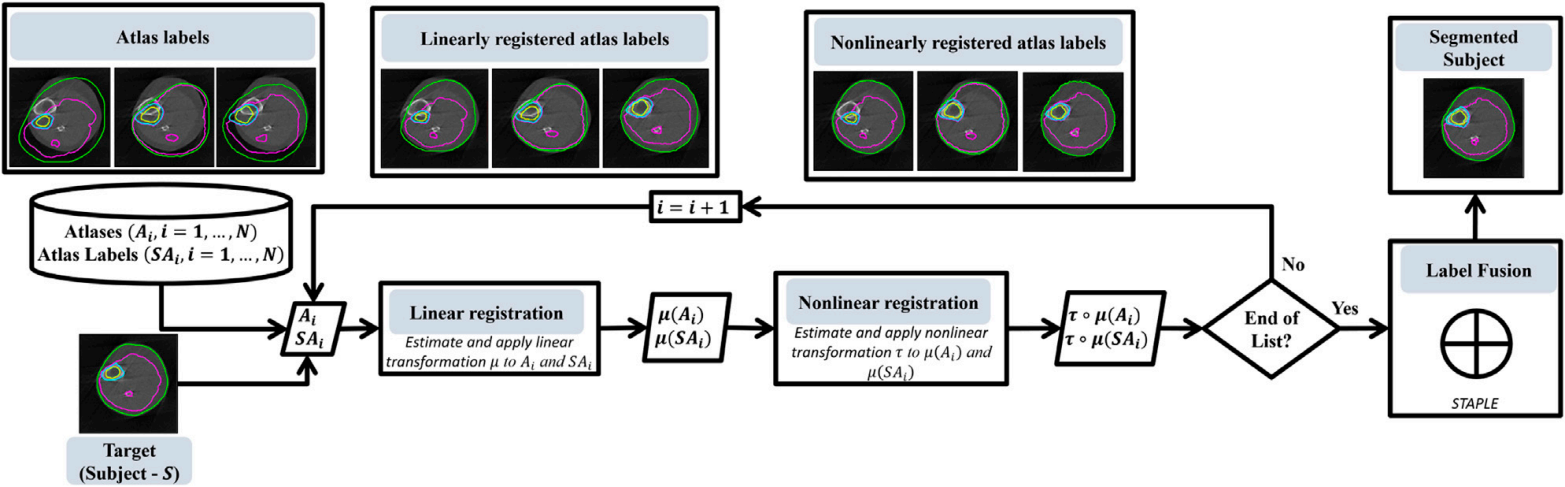
Flowchart that shows the main stages of our multi-atlas based segmentation methodology.

In multi-atlas image segmentation (MAIS), multiple atlases *A*_*i*_, *i* = 1, *…* , *N* and corresponding segmented atlases *SA*_*i*_, *i* = 1, *…* , *N* are used to produce a segmentation of the subject *S*. The main stages of a multi-atlas-based segmentation algorithm are *registration, label propagation* and *label fusion*. Figure 3 shows the main stages of MAIS. In the following subsections, we elaborate on our method based on these stages. We will use *A* when referring to each atlas *A*_*i*_, *i* ∈ {1, *…* , *N*}.

### 2.2 Image registration

Image registration is a key stage of atlas-based segmentation as described in the previous section. The goal of registration is to align the spatial domain of a subject with that of an atlas. In other words, we wish to find the optimal deformation τ* in the set {*τ*, *τ* : (*A*, Ω_*A*_) → (*S*, Ω_*S*_)} of all transformations from the spatial domain of the source image (or atlas) (*A*, Ω_*A*_) to the subject space (*S*, Ω_*S*_), that minimizes the following energy functional (Sotiras et al., 2013; Vercauteren et al., 2007),

$$E\left(\tau \right)≔\lambda_{\zeta }\zeta \left(S,A◦\tau \right)+\lambda_{\rho }\rho \left(\tau \right),$$ (1)

where *ζ* is the similarity term, *ρ* is the regularization term, *λ*_*ζ*_ and *λ*_*ρ*_ are the weights of the similarity and regularization terms, respectively. Therefore,

$$\tau^{*}=\arg\underset{\tau }{\min}E\left(\tau \right).$$ (2)

To ensure accurate registration we applied both linear and nonlinear registrations. Linear registration is used to capture the rigid displacement of the subject while nonlinear registration is used to capture the local deformation of the anatomical structures of the subject. Anatomical structures are the tissue types, i.e., the trabecular bone, cortical bone, muscle, and subcutaneous adipose tissue as shown in Table 1. Figure 2 shows examples of rigid displacements of atlas *A* denoted by *μ*(*A*), and local deformations *τ*◦*μ* produced by nonlinear registration *τ*◦*μ*. Our techniques for linear and nonlinear registration are described below.

**TABLE 1:** Tissues to quantify in each anatomical site.

Anatomical site	Tissues to quantify
4% tibia	Trabecular Bone (TB)
38% tibia	Cortical Bone (CB), Trabecular Bone
66% tibia	Cortical Bone, Trabecular Bone, Subcutaneous Adipose Tissue (SAT), Muscle

#### 2.2.1 Linear registration

Given a subject *S* and for each atlas *A*, we estimate the parameters of a linear transformation *μ* from atlas space (*A*, Ω_*A*_) to the subject space (*S*, Ω_*S*_) that defines the rigid motion between the atlas and the subject. Our linear transformation is modeled using affine transformations. We utilize the Mattes’ mutual information similarity measure (Mattes et al., 2003) given in Eq. 3,

$$\zeta \left(S,A◦\mu \right)=-\underset{\iota }{\sum }\underset{\kappa }{\sum }p\left(\iota ,\kappa |\mu \right)\log\frac{p\left(\iota ,\kappa |\mu \right)}{p_{A}\left(\iota |\mu \right)p_{S}\left(\kappa \right)}$$ (3)

and a regular step gradient descent optimizer, to estimate the parameters of the affine transformation *μ*,where *p*, is the joint probability distribution of subject and the atlas, *p*_*A*_ and *p*_*S*_ are the marginal probability distributions of the atlas and the subject respectively, *ι* = 1, *…* , *n*_*A*_ and *κ* = 1, *…* , *n*_*S*_ are the indices of the histogram bins for the source and target image. The image *μ*(*A*) of the atlas *A* lives in the subject space and approximates the rigid motion between the subject and the atlas (Figure 2).

#### 2.2.2 Nonlinear registration

In this stage, the goal is to correct the local deformations between the subject and the atlas. Hence we seek the parameters of deformation *τ* : (*μ*(*A*), Ω_*S*_) → (*S*, Ω_*S*_) from *μ*(*A*) ⊂ *S* onto *S*. We introduce the use of three nonlinear deformable registration techniques, free-form deformation (FFD), symmetric diffeomorphic demons (SDD), and symmetric image normalization (SyN, SyNO) for our multi-atlas-based registration. We discuss the nonlinear methods for our multi-atlas-based image segmentation below.

##### 2.2.2.1 Free-form deformations

Free-form deformation was originally proposed by Rueckert et al., 1999 and was applied to automated registration of breast MRI scans. It uses a spline interpolation kernel to compute the deformation values between the control points that produces a locally controlled, globally smooth transformation.

Given a 2D spatial domain Ω = {**x** = (*x*, *y*)|0 ≤ *x* < *X*, 0 ≤ *y* < *Y*} of an image, let Φ denote an *n*_*x*_ × *n*_*y*_ mesh of control points *ϕ*_*i*,*j*_= (*iδ*, *jδ*) with uniform spacing *δ*. Rueckert et al., 1999 proposed a method that seeks the optimal FFD (displacement field *τ*) written as the 2-D tensor product of 1-D cubic B-Splines

$$\tau \left(x\right)=\overset{2}{\underset{l=0}{\sum }}\overset{2}{\underset{m=0}{\sum }}B_{l}\left(u\right)B_{m}\left(v\right)\phi_{i+l,j+m}$$ (4)

that optimizes the energy functional in Eq. 1. In Eq. 4, *i* = ⌊*x*/*n*_*x*_⌋ − 1, *j* = ⌊*y*/*n*_*y*_⌋ − 1, *u* = *x*/*n*_*x*_ − ⌊*x*/*n*_*x*_⌋, *v* = *y*/*n*_*y*_ − ⌊*y*/*n*_*y*_⌋, and *B*_*l*_ represents the *l*th basis function of the B-spline. The regularization term *ρ* is given by the bending energy of a thin-plate of metal, which controls the smoothness of the transformation, defined by

$$\rho \left(\tau \right)=\frac{1}{\left|\Omega \right|}\iint_{\Omega }{\left(\frac{\partial \tau }{\partial x}+\frac{\partial \tau }{\partial y}\right)}^{2}d\Omega ,$$ (5)

where |Ω| is the cardinality of the image spatial domain. We employed Matte’s mutual information (Mattes et al., 2003) defined in Eq. 3 as the similarity measure *ζ*. We used *λ*_*ζ*_ = −1 and *λ*_*ρ*_ = 0.01 in the energy function of Eq. 1. The optimization process is based on updating control points *via* the gradient of the cost function. We employed Limited-memory Broyden–Fletcher–Goldfarb–Shannon optimization to find the energy minimum of Eq. 1. We embedded this method in a hierarchical multi-scale structure to be able to capture a wide range of deformations. This structure contains a coarse and fine scale for optimization. At the coarse scale, the optimizer can capture extensive deformations, but the solution may have limited precision. So we use the coarse solution to initialize the optimization at the fine scale and find a more precise deformation field.

##### 2.2.2.2 Symmetric diffeomorphic demons

We utilize a variant of the Demons algorithm that is optimized in the log domain as proposed in Vercauteren et al., 2008. This is a variational method that seeks to minimize the following energy functional:

$$E\left(c,\tau \right)=\frac{1}{\lambda_{\zeta }^{2}}\frac{1}{2|\Omega_{p}|}\underset{x\in \Omega_{x}}{\sum }|S\left(x\right)-A\left(\tau \left(x\right)\right){|}^{2}+\frac{1}{\lambda_{h}^{2}}\|\log\left(\tau^{-1}◦c\right){\|}^{2}+\frac{1}{\lambda_{\rho }^{2}}\|\nabla \log\left(\tau \right){\|}^{2}$$ (6)

where the variable *c* was introduced to approximate the error in the correspondence between image pixels, *λ*_*h*_ accounts for spatial uncertainty on the correspondences, and Ω_**p**_ is the region of overlap between *S* and *A*◦*τ*.

In the update step, and under the assumption that the current transformation *τ* is expressible as an exponential of smooth vector fields **v**, i.e., *τ* = exp(**v**), the Baker-Campbell Hausdorff (BCH) approximations are used to seek a smooth velocity field *Z* (**v**, *ɛ* **u**), such that $\exp\left(Z\right.\left(v,\varepsilon u\right)\approx \tau ◦\exp\left(\varepsilon u\right)$ , where *ɛ* is a weight parameter. Then **u** is given by

$$u\left(x\right)=-\left(\frac{\left(S\left(x\right)-A◦\tau \left(x\right)\right)}{\left(\|J^{p}{\|}^{2}+\left(\lambda_{\zeta }^{2}\left(x\right)/\lambda_{h}^{2}\right)\right)}\right)J^{x^{⊤}},$$ (7)

and *J* is the Jacobian matrix. In the log-domain, the inverse of a spatial transform *τ* ^−1^, parametrized by *τ* = exp(**v**), can be obtained efficiently by backward computation *τ* ^−1^ = exp (−**v**). A symmetric transformation can be obtained from a nonsymmetric one by making the global energy symmetric, i.e.,

$$\tau_{\mathrm{opt}}=\arg\underset{\tau }{\min}\left(E_{\mathrm{sym}}≔E\left(S,A◦\tau \right)+E\left(A,S◦\tau^{-1}\right)\right).$$ (8)

The minimization of the energy functional in Eq. 8 can be formulated as a constrained equation using two diffeomorphisms

$$\left[\tau_{\mathrm{opt}},\tau_{\mathrm{opt}}^{-1}\right]=\arg\underset{\left[\tau ,\tau^{-1}\right]}{\min}E_{\mathrm{sym}}.$$ (9)

##### 2.2.2.3 Symmetric image normalization

This method uses diffeomorphisms as the transformation model (Avants et al., 2008). SyN performs the normalization by minimizing the energy functional defined in Eq. 10. SyN searches for a symmetric diffeomorphic spatiotemporal mapping, *τ* ∈ *Diff*_0_: = {the space of diffeomorphic mappings with homogeneous boundary conditions} that minimizes the energy functional in the optimization problem defined in Eq. 10.

$$\begin{array}{c} E_{\mathrm{sym}}\left(S,A\right)=\underset{\tau_{1}}{\inf}\underset{\tau_{2}}{\inf}\int_{t=0}^{\frac{1}{2}}\left\{\|\nu_{1}\left(\tau_{1}\left(x,t\right),t\right){\|}_{L}^{2}+\|\nu_{2}\left(\tau_{2}\left(x,t\right),t\right){\|}_{L}^{2}\right\}dt+\int_{\Omega }\zeta \left(S\left(\tau_{2}\left(0.5\right)\right.\right.,A\left(\tau_{1}\left(0.5\right)\right)d\Omega \\ \text{ subject to each }\tau \in Diff_{0}\text{ the solution of: }d\tau \left(x,t\right)/dt=\nu_{i}\left(\tau_{i}\left(x,t\right),t\right)\\ \text{ with }\tau_{i}\left(x,0\right)=I\text{, and }\tau_{i}^{-1}\left(\tau_{i}\right)=I,\;\tau_{i}\left(\tau_{i}^{-1}\right)=I \end{array}$$ (10)

The first integral in Eq. 10 corresponds to the regularization term that is induced by a functional norm ‖⋅‖_*L*_ through a linear differential operator *L* = *a*∇^2^ + *b* **I**, with constants *a* and *b*, and **I** is the identity mapping. The second integral corresponds to the similarity term between the reference and input image, where *ζ* is Mattes’ mutual information defined in Eq. 3, Ω is the common spatial domain of the images, *ν*(**x**, *t*) is the velocity field, and *t* is the time. The optimization process performs gradient descent to update the deformation field and a fixed point method calculates the inverse transformation. The velocity fields *ν*_*i*_ are computed iteratively, and they update the deformation *τ*_*i*_, *i* = 1, 2. The deformable registration stage is preceded by rigid and affine transformation steps to address global misalignments as described in.

### 2.3 Shape modeling - statistical atlas generation

We generate the statistical atlases by iteratively averaging subject scans that are mapped onto a common reference space. We first choose one subject to serve as the reference scan. Then, we linearly register all subjects to the selected reference and compute the group average. In the second iteration, we use as reference the average image produced by linear group registration. Next, we map all subjects to the new computed template using nonlinear registration, and compute the average. In the remaining iterations we only apply nonlinear mappings to update the average image. We repeat the above steps until the final iteration *i* = *K*. This process converged to an atlas template within *K* = 5 iterations on our data.

The above steps generate sequences of transformations $\tau_{i}^{\left(n\right)}$ , where *n* = 0, 1, 2, *…* , *N* is the *n*th image and *i* = 0, 1, *…* , *K* is the *i*th iteration. Rohlfing et al., 2001 showed that in each iteration *i*, the transformation $\tau_{i}^{\left(n+1\right)}$ and the preceding transformation $\tau_{i}^{\left(n\right)}$ differ only by a small amount of deformation. Finally, a human operator labeled the trabecular bone, cortical bone, muscle, and SAT using the MIPAV software suite (McAuliffe et al., 2001) by manual selection of control points and spline interpolation. In the first row of Figure 2, we display our computed atlas image and the atlas label map for 4%, 38% and 66% of the tibia length, respectively. In the first row, third, sixth and ninth columns of Figure 2, we display the color-coded atlas labels, for each tibial site under consideration.

### 2.4 Multi-atlas based tissue segmentation

#### 2.4.1 Label propagation

Label propagation is the process of assigning labels from the warped atlas labels to the reference space. We use the linear (*μ*) and nonlinear (*τ*) transformations between *S* and *A* that we found in the registration stage, to map labels from the atlas to the subject space. Label propagation is achieved by nearest neighbor interpolation after warping the atlas label to the subject domain *via* the estimated deformation *τ*≔*τ*◦*μ*. The segmentation map is produced by *τ*(*SA*).

#### 2.4.2 Label fusion

This is a key stage of MAIS. Here, we combine all the propagated atlases to obtain a final segmentation (Iglesias and Sabuncu 2015). Various methods have been proposed for this stage including best atlas selection, a selective and iterative method for performance level estimation (SIMPLE), joint label fusion, majority voting, weighted majority voting, and simultaneous truth and performance level estimation (STAPLE) algorithm (Warfield et al., 2004; Langerak et al., 2010; Wang et al., 2013; Iglesias and Sabuncu 2015). In this work, we utilized STAPLE for fusing segmentation results by individual atlases. We utilize STAPLE for label fusion because it has performed very well over a range of applications Cardoso et al., 2013; Weston et al., 2019.

STAPLE can be formulated using probabilistic classification terms. Given *K* segmentations (classifications) of a subject *S* having *N* pixels, let *e*_*k*_(**x**) be the decision of classifier *k* at voxel **x**. If the (unknown) ground truth label for voxel **x** is *l*, we say that **x** ∈ *C*_*l*_. The performance of classifier *k* is determined by two parameters *p* (sensitivity) and *q* (specificity), referring to the fractions of true positives and true negatives among the classified voxels, that maximizes the complete log likelihood function, (*p*, *q*) = arg max_*p*,*q*_ ln *f* (*D*, *T*|*p*, *q*), where $T=\cup_{l=1}^{n}C_{l}$ is the true segmentation, also called missing or hidden data, and *D* = [*e*_*k*_(**x**)] is an *N* × *K* decision matrix.

For each classifier *k*, and each class *C*_*l*_, the parameters *p* and *q* are modeled independently as the following conditional probabilities: *p*_*k*_ = Pr (*e*_*k*_(**x**) = *l*|*x* ∈ *C*_*l*_)and *q*_*k*_ = Pr (*e*_*k*_(**x**) ≠ *l*|**x**∉*C*_*l*_). The process of estimation of *p* and *q* is achieved by the Expectation-Maximization (EM) algorithm.

The final segmentation $\hat{S}$ at voxel **x** is computed by *E*(**x**) = arg max_*i*_ *P* (*x* ∈ *C*_*i*_|*e*_1_(**x**), *…*, *e*_*K*_(**x**)). The probability *p* (**x** ∈ *C*_*i*_|*e*) follows from the classifier’s decisions and their performance parameters using Bayes’ rule (Rohlfing et al., 2005).

## 3 Data description and performance evaluation measures

### 3.1 Overview of dataset

We used pQCT data obtained from the InCHIANTI clinical study to evaluate the performance of the methods. InCHIANTI is a longitudinal study of risk factors for mobility disability performed in a representative sample of the middle aged and older populations living in Tuscany, Italy (Ferrucci et al., 2000; Lauretani et al., 2008; Makrogiannis et al., 2018). The validation dataset is randomly sampled from the original InCHIANTI baseline database as in Makrogiannis et al., 2018. It consists of pQCT scans of the lower leg acquired at the 4%, 38% and 66% tibial length of the lower leg. Our randomly sampled dataset contains a total of 77 samples, that is 30 samples at 4%, 27 samples at 38% and 20 samples at 66% tibial length. The pQCT scans of the right lower leg were acquired using a XCT 2000 scanner (Stratec Medizintechnik GmbH, Pforzheim, Germany). The slice thickness of each scan is 2.1 *mm* and the in-plane slice is 0.5 *mm*. At each tibial location, we used three templates for MAIS. One of these three samples was used to initialize statistical atlas generation. We cross-validated the performance of each method on the testing samples that remained after removing the three templates. We focused on specific tissue(s) of interest at each tibial location: trabecular bone at 4%; cortical and trabecular bone at 38%; subcutaneous adipose tissue (SAT), muscle, cortical bone and trabecular bone at 66% as shown in Table 1.

### 3.2 Motion artifacts

Unique characteristics of the test data that made it suitable for our analysis and clinically relevant are: 1) the strong representation of older persons who are likely to experience walking difficulties, 2) the different locations of the tibial length that we considered for identification of six different tissue types (Table 1), 3) the incidence of motion artifacts in the dataset making it complicated for segmentation. Motion artifacts occur as a result of the voluntary and involuntary movement of the subject during image acquisition.

Frequent criteria for motion assessment are discontinuities and streaks in the cortical bone, and blurring and shifting of trabecular bone. A clinical specialist used the above visual inspection criteria and a grading system from 1 to 5 (1: no artifacts) to classify the subject motion artifacts as described in Wong 2016. This effect was more evident at the 66% tibial length with more than 50% of the samples having significant motion artifacts corresponding to grades 4 and 5.

### 3.3 Validation

We performed quantitative analysis of the performance of the atlas-based tissue identification schemes by calculating the Dice Similarity Coefficient (DSC), sensitivity or True Positive Rate (TPR), and Precision (PR) between segmentation results and their ground truths *T*. We cross-validated the performance metrics by excluding the two subjects we used as templates in MAIS, and the subject we used as template for statistical atlas generation. We made comparisons across single- and multi-atlas based image segmentation methods with respect to registration algorithms. We compared the results obtained by multi-atlas- based techniques with those obtained by the automated tissue identification and quantification (TIDAQ) method (Makrogiannis et al., 2018), to emphasize the performance of MAIS techniques. We chose TIDAQ for our comparisons because it is a model-based tissue segmentation method that has produced good results for images of good quality, but may be challenged by images that have moderate to significant motion artifacts. We performed nonparametric Wilcoxon rank sum tests to examine the effects of motion artifacts on tissue identification using pQCT scans of 66% tibia. The sample size is *n* = 20.

### 3.4 Tissue quantification

We provide an extension of our analysis to non-labeled pQCT scans of the lower leg from the InCHIANTI dataset to quantify body composition changes caused by aging. Our aim is to show the reliability of the MAIS-technique using SDD-STPL for tissue quantification on an extended clinical dataset and evaluate the agreement of our results with clinical observations. We decided to quantify the baseline InCHIANTI dataset that includes a total of 2,425 pQCT scans. We applied the following procedures to prepare the data for analysis. With the help of TIDAQ software, we sorted the scans according to anatomical sites, selected scans at 4%, 38%, and 66% of tibial length and removed scans with different orientations. We then separated the remaining scans according to their gender (male and females). After data preparation, we had a total of 1748 scans for quantification that may be grouped as follows: 585 scans (males: 272, and females: 313) at 4%; 583 scans (males: 272, females: 311) at 38%; and 580 scans (Males: 270, females: 304) at 66%. A summary of our quantification dataset, including gender and age distribution, is provided in Table 2.To characterize the effect of aging on body composition, we calculated the cross-sectional area (*CSA*) and density of the trabecular bones at 4% tibia, cortical bones at 38% tibia, and muscle and subcutaneous adipose tissue (SAT) at 66% tibia. To obtain these measures, we ran our SDD-STPL technique on all datasets to automatically identify these tissues and computed the total CSA and the average density for each tissue type. We decided to perform analyses of both genders jointly, as well as separate gender-conditional analyses.

**TABLE 2:** Summary of the unlabeled pQCT scans of the lower leg from the InCHIANTI Study.

Site (%)	Gender	*N*	Age (yrs)				
			*Min*	*Max*	*Med*	*Ave*	*Std*
4	Male	272	28	104	74	87.3	14.2
	Female	313	26	104	74	87.4	15.0
	Total	585	26	104	73	87.4	14.6
38	Male	272	28	104	73	69.2	14.5
	Female	311	26	104	74	69.4	14.7
	Total	583	26	104	74	69.3	14.6
66	Male	270	28	104	73	69.2	14.3
	Female	304	26	104	73	69.1	14.8
	Total	580	26	104	73	69.1	14.5

We used scatter plots and regression analyses of quantification results to study the changes in body composition (response variable) with respect to age (predictor variable). We utilized the following statistical measures to analyze the relationship between the two variables: the square of the correlation coefficient (*R* ^2^), the coefficient of variation of the root mean squared difference (*CV*-*RMSD*) (Eq. 11) between the reference (*y*) and the predicted $\left(\hat{y}\right)$ measurements, the slope and intercept of the regression line, and the *p* − values.

$$CV-RMSD=\frac{1}{\mu }\sqrt{\frac{\sum_{k=1}^{N}{\left(y_{k}-{\hat{y}}_{k}\right)}^{2}}{N}};\;\;\mu =\frac{\sum_{k=1}^{N}y_{k}}{N}$$ (11)

## 4 Experiments and results

Here we evaluate the performances of the four MAIS techniques and an automated model-based tissue quantification method (TIDAQ) (Makrogiannis et al., 2018). We then explore the effect of subject movement artifacts on tissue segmentation performance. Finally, we expand our analysis to quantify the complete InCHIANTI baseline dataset.

Our aim is to support or reject the hypotheses that 1) MAIS techniques, in general, improve the segmentation performance of SAIS, 2) STAPLE on SDD mappings produces better segmentation quality than the other methods, 3) MAIS is more resilient to subject movement than model-based segmentation.

We analyzed the performance of the deformable methods by validating the SAIS results of statistical atlases against reference standards, *T*. Reference standard is a tissue label map that was generated manually by a clinical specialist. We evaluated single atlas segmentation performances of STAT-FFD, STAT-SDD, STAT-SyN, and STAT-SyNO, where ‘STAT’ represents the statistical atlas. The MAIS techniques we developed and evaluated in this framework are STPL-FFD, STPL-SDD, STPL-SyN, and STPL-SyNO. ‘SyNO’ denotes an ‘Symmetric Normalization with our own linear registration’ and ‘STPL’ denotes ‘STAPLE.’

### 4.1 4% tibia segmentation

**FIGURE 4:**
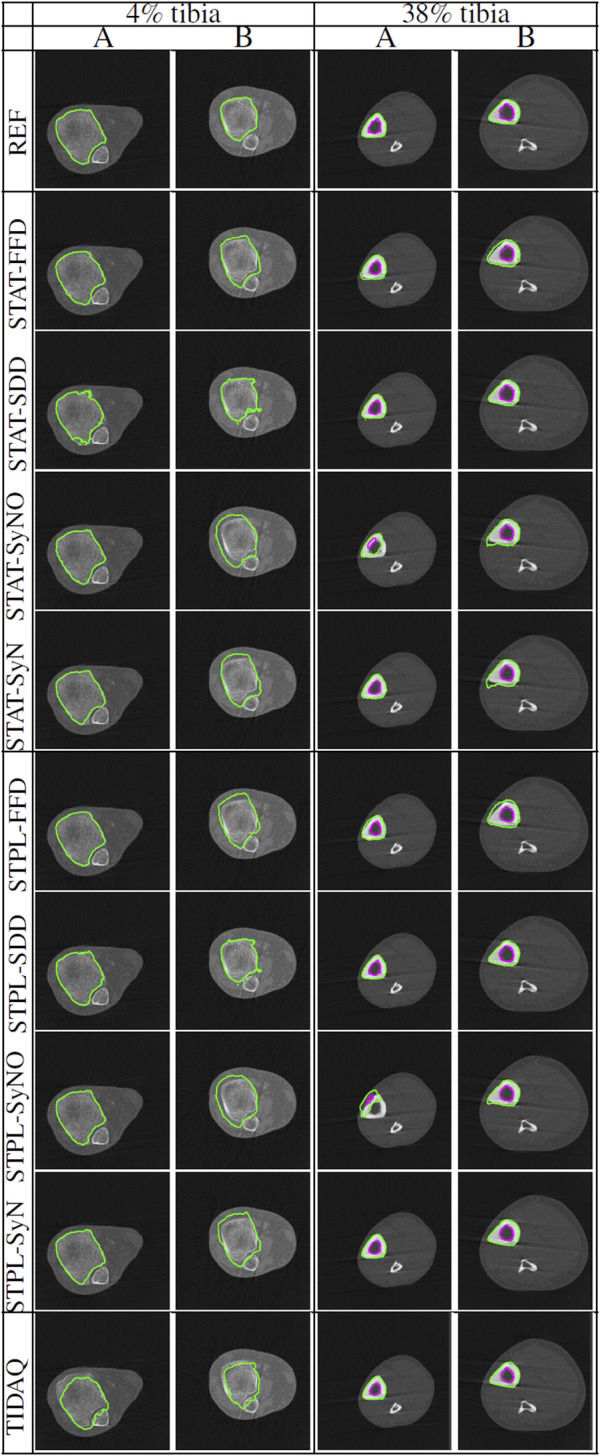
Comparisons of tissue segmentation by MAIS and model-based methods at 4% tibia and 38% tibia. Two subjects (subject **(A)** and subject **(B)**) are selected at each tibial site to demonstrate the performance of the compared methods. At 4% the trabecular bone is delineated by the green contour, and at 38% the cortical bone and the trabecular bone are delineated by the green and magenta contours, respectively.

Table 3 displays the summarized performance measures obtained by the single-atlas image segmentation using the statistical atlas and multi-atlas image segmentation methods, and TIDAQ for identification of the trabecular bone (TB) over the test-set. In Figure 4, column 1 we show examples of multi-atlas segmentations of the trabecular bone at 4% tibia by the compared methods, delineated by the green contours.

**TABLE 3:** 4% Trabecular Bone Segmentation Performance (mean ± standard deviation). DSC: Dice Similarity Coefficient, TPR: True Positive Rate, PR: Precision.

Method	DSC	TPR	PR
STAT-FFD	0.947 ± 0.045	0.96 ± 0.021	0.939 ± 0.084
STPL-FFD	0.936 ± 0.056	0.971 ± 0.018	0.911 ± 0.103
STAT-SDD	0.959 ± 0.019	0.939 ± 0.037	0.981 ± 0.015
STPL-SDD	**0.972 ± 0.009**	0.965 ± 0.021	**0.98 ± 0.014**
STAT-SyNO	0.906 ± 0.072	0.922 ± 0.081	0.9 ± 0.103
STPL-SyNO	0.914 ± 0.068	0.936 ± 0.07	0.902 ± 0.104
STAT-SyN	0.947 ± 0.05	0.972 ± 0.017	0.93 ± 0.122
STPL-SyN	0.947 ± 0.054	**0.975 ± 0.02**	0.928 ± 0.128
TIDAQ	0.941 ± 0.022	0.913 ± 0.038	0.972 ± 0.024

A comparison of single-atlas and multi-atlas segmentation results in Table 3 shows that the use of multiple atlases improves segmentation quality. With respect to multi-atlas image segmentation techniques, we examined the performance quality of the methods and the effect of label fusion on the improvement of the results. The range and mean ± standard deviation of DSC values produced by STPL-SDD are [0.944, 0.992] and 0.972 ± 0.009, by STPL-FFD are [0.739, 0.991] and 0.936 ± 0.056, by STPL-SyN are [0.743, 0.990] and 0.947 ± 0.054, and by STPL-SyNO are [0.731, 0.991] are 0.914 ± 0.068, respectively, as can be seen in Table 3. In summary, we observe that STPL-SDD outperformed the other multi-atlas techniques.

The range of DSC values produced by TIDAQ for trabecular bone identification is [0.897, 0.967] with mean ± standard deviation of 0.941 ± 0.022. The results in Table 3 indicate that TIDAQ outperformed STPL-FFD, and STPL-SyNO registration techniques. On the other hand, TIDAQ was less accurate than STPL-SDD, and STPL-SyN.

### 4.2 38% tibia segmentation

Tables 4 and 5 contain the performance measures produced by these experiments. Figure 4 displays examples of tissue delineations, on two of our test subjects.

**TABLE 4:** 38% Cortical Bone Segmentation Performance (mean ± standard deviation). DSC: Dice Similarity Coefficient, TPR: True Positive Rate, PR: Precision.

Method	DSC	TPR	PR
STAT-FFD	0.809 ± 0.126	0.933 ± 0.064	0.721 ± 0.125
STPL-FFD	0.781 ± 0.136	0.889 ± 0.135	0.706 ± 0.153
STAT-SDD	0.932 ± 0.022	0.965 ± 0.025	0.902 ± 0.032
STPL-SDD	**0.947 ± 0.021**	0.949 ± 0.036	**0.947 ± 0.023**
STAT-SyNO	0.78 ± 0.174	0.891 ± 0.173	0.704 ± 0.184
STPL-SyNO	0.76 ± 0.215	0.841 ± 0.233	0.704 ± 0.216
STAT-SyN	0.871 ± 0.07	**0.983 ± 0.026**	0.787 ± 0.104
STPL-SyN	0.873 ± 0.112	0.96 ± 0.079	0.807 ± 0.141
TIDAQ	0.897 ± 0.15	0.954 ± 0.156	0.847 ± 0.147

**TABLE 5:** 38% Trabecular Bone Segmentation Performance (mean ± standard deviation). DSC: Dice Similarity Coefficient, TPR: True Positive Rate, PR: Precision.

Method	DSC	TPR	PR
STAT-FFD	0.823 ± 0.084	0.774 ± 0.145	0.917 ± 0.121
STPL-FFD	0.797 ± 0.131	0.759 ± 0.177	0.891 ± 0.155
STAT-SDD	0.92 ± 0.027	0.868 ± 0.052	**0.981 ± 0.019**
STPL-SDD	**0.939 ± 0.019**	**0.91 ± 0.041**	0.972 ± 0.026
STAT-SyNO	0.703 ± 0.294	0.627 ± 0.282	0.831 ± 0.309
STPL-SyNO	0.687 ± 0.343	0.624 ± 0.338	0.807 ± 0.363
STAT-SyN	0.849 ± 0.079	0.764 ± 0.081	0.97 ± 0.109
STPL-SyN	0.877 ± 0.08	0.811 ± 0.11	0.972 ± 0.078
TIDAQ	0.825 ± 0.167	0.726 ± 0.156	0.96 ± 0.187

Overall, quantitative results of the methods reported in Tables 4 and 5, show that SDD produced better segmentation quality than the other deformable models, followed by SyN. Low DSC values are produced by SyN, FFD, and SyNO models (in decreasing order) for both cortical and trabecular bone in some subjects. SyNO missed the trabecular bone in few subjects producing zero DSC. We also noticed that all deformable methods produced DSC means that are greater than 75% for cortical and trabecular bone except SyNO in the trabecular bone. We infer that the range of DSC values for SDD is more compact than the other deformable models. The minimum value of DSC produced by SDD is greater than 85% for cortical bone, and about 82% for trabecular bone. The DSC values produced by SyN are fairly compact in the identification of bone compartments, and their mean values are greater than 85% with standard deviations about 10%.

In Tables 4 and 5, we observe that FFD results for both single- and multi-atlas image segmentation techniques show a wider DSC dispersion than SDD, the former producing values lower than 60% for cortical bone and about 45% for trabecular bone.

SyNO results show wider DSC spread than all of the other deformable models. SyNO produced the least mean DSC of about 78% for identification of cortical bone and about 68% for trabecular bone. This error is usually caused by linear registration failures propagated to the symmetric normalization stage.

TIDAQ performed very well in the identification of trabecular bone, and much better in the identification of cortical bone. Overall, the identification accuracy is promising with DSC mean ± standard deviation of 89.7 ± 2.2% for cortical bone and 82.5 ± 16.7% for trabecular bone. Despite the good performance of TIDAQ, STPL-SDD outperformed it in the identification of cortical bone, while STPL-SDD and STPL-SyN outperformed it in the identification of trabecular bone. This shows that multi-atlas image segmentation techniques have the potential to produce higher tissue identification accuracy than TIDAQ.

### 4.3 66% tibia segmentation

**FIGURE 5:**
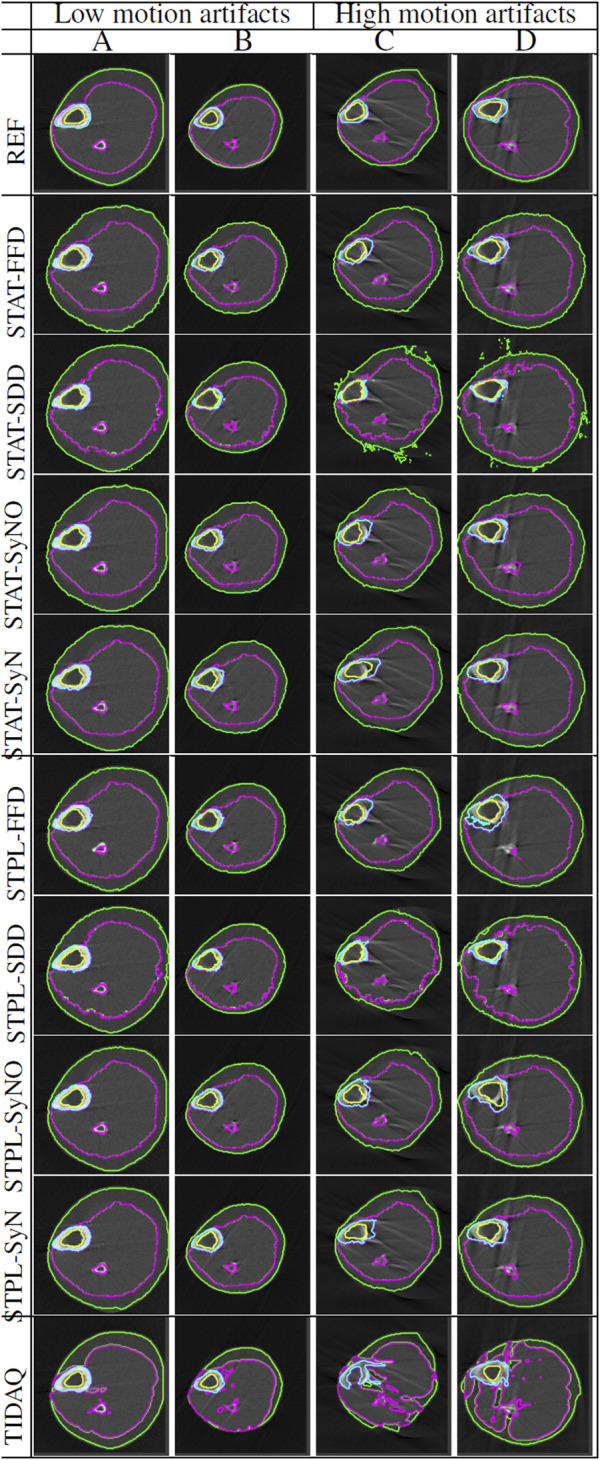
Segmentation comparisons of scans at 66% tibial length with low and high levels of artifacts caused by subject motion. Scans **(A,B)** have low motion artifacts, while scans **(C,D)** have high motion artifacts. The delineation of the subcutaneous adipose tissue (SAT) is represented by the green contour, muscle by magenta color, cortical bone by cyan color, and trabecular bone by yellow color.

Figure 5 displays segmentation results produced by the compared approaches on two scans with low motion degradation and two scans with high motion degradation. The mean ± standard deviation values of DSC, TPR and PR of each tissue over all testing samples are given in Tables 6–9. Overall, SDD exhibited better performance across the three metrics than the other deformable models for the identification of all tissues, followed by SyN and TIDAQ. In addition, SDD produced DSC, true positive rate, and precision values of lower dispersion (expressed by smaller standard deviation) than the other methods. STPL-SDD yielded the top DSC performance for SAT, muscle, and trabecular bone segmentation. The DSC minimum values for this method were about 62.7% for SAT, 89% for muscle, 75% for cortical bone, and 86.2% for trabecular bone. All MAIS techniques produced mean DSC greater than 89%, mean TPR greater than 84%, and mean PR greater than 95% in muscle segmentation. Conversely, all tested methods yielded mean DSC less than 80%, and a top PR of 70.2% in SAT segmentation.

**TABLE 6:** 66% SAT Segmentation Performance (mean ± standard deviation). DSC: Dice Similarity Coefficient, TPR: True Positive Rate, PR: Precision.

Methods	DSC	TPR	PR
STAT-FFD	0.682 ± 0.158	**0.953 ± 0.051**	0.562 ± 0.2
STPL-FFD	0.73 ± 0.16	0.912 ± 0.085	0.649 ± 0.227
STAT-SDD	0.708 ± 0.151	0.939 ± 0.042	0.594 ± 0.189
STPL-SDD	**0.771 ± 0.144**	0.917 ± 0.057	0.692 ± 0.202
STAT-SyNO	0.684 ± 0.152	0.938 ± 0.065	0.572 ± 0.2
STPL-SyNO	0.729 ± 0.161	0.892 ± 0.093	0.659 ± 0.229
STAT-SyN	0.677 ± 0.147	0.935 ± 0.079	0.565 ± 0.197
STPL-SyN	0.734 ± 0.157	0.889 ± 0.078	0.664 ± 0.225
TIDAQ	0.746 ± 0.228	0.845 ± 0.14	**0.702 ± 0.267**

**TABLE 7:** 66% Muscle Segmentation Performance (mean ± standard deviation). DSC: Dice Similarity Coefficient, TPR: True Positive Rate, PR: Precision.

Methods	DSC	TPR	PR
STAT-FFD	0.893 ± 0.032	0.837 ± 0.067	0.962 ± 0.037
STPL-FFD	0.902 ± 0.034	0.857 ± 0.074	0.959 ± 0.037
STAT-SDD	0.922 ± 0.029	0.891 ± 0.057	0.958 ± 0.032
STPL-SDD	**0.938 ± 0.028**	**0.914 ± 0.057**	**0.966 ± 0.029**
STAT-SyNO	0.894 ± 0.038	0.847 ± 0.073	0.953 ± 0.054
STPL-SyNO	0.911 ± 0.038	0.874 ± 0.078	0.959 ± 0.048
STAT-SyN	0.893 ± 0.035	0.844 ± 0.077	0.957 ± 0.051
STPL-SyN	0.913 ± 0.04	0.875 ± 0.084	0.962 ± 0.045
TIDAQ	0.904 ± 0.085	0.861 ± 0.131	0.963 ± 0.025

**TABLE 8:** 66% Cortical Bone Segmentation Performance (mean ± standard deviation). DSC: Dice Similarity Coefficient, TPR: True Positive Rate, PR: Precision.

Methods	DSC	TPR	PR
STAT-FFD	0.665 ± 0.152	0.668 ± 0.121	0.677 ± 0.196
STPL-FFD	0.668 ± 0.132	0.696 ± 0.11	0.666 ± 0.19
STAT-SDD	0.681 ± 0.094	0.55 ± 0.112	**0.911 ± 0.059**
STPL-SDD	0.829 ± 0.083	0.776 ± 0.118	0.898 ± 0.064
STAT-SyNO	0.721 ± 0.156	0.708 ± 0.145	0.754 ± 0.201
STPL-SyNO	0.765 ± 0.194	0.792 ± 0.181	0.761 ± 0.225
STAT-SyN	0.718 ± 0.147	0.719 ± 0.084	0.737 ± 0.21
STPL-SyN	0.788 ± 0.152	0.827 ± 0.112	0.769 ± 0.194
TIDAQ	**0.851 ± 0.115**	**0.987 ± 0.032**	0.76 ± 0.157

**TABLE 9:** 66% Trabecular Bone Segmentation Performance (mean ± standard deviation). DSC: Dice Similarity Coefficient, TPR: True Positive Rate, PR: Precision.

Methods	DSC	TPR	PR
STAT-FFD	0.825 ± 0.087	0.902 ± 0.124	0.783 ± 0.124
STPL-FFD	0.827 ± 0.085	0.911 ± 0.123	0.783 ± 0.138
STAT-SDD	0.86 ± 0.049	**0.983 ± 0.023**	0.769 ± 0.08
STPL-SDD	**0.913 ± 0.045**	0.968 ± 0.037	0.869 ± 0.088
STAT-SyNO	0.843 ± 0.179	0.91 ± 0.203	0.803 ± 0.142
STPL-SyNO	0.868 ± 0.167	0.913 ± 0.191	0.838 ± 0.14
STAT-SyN	0.856 ± 0.116	0.946 ± 0.073	0.791 ± 0.149
STPL-SyN	0.895 ± 0.105	0.939 ± 0.077	0.862 ± 0.133
TIDAQ	0.871 ± 0.221	0.816 ± 0.216	**0.994 ± 0.026**

In SAT delineation, FFD and SyNO models compete with each other in the identification of SAT, but SyNO outperforms free-form deformation in the identification of other tissues by at least 10% accuracy. Except for cortical bone, we observe that MAIS outperformed TIDAQ.

### 4.4 Effects of subject motion on tissue identification at 66% tibia

In this experiment, we studied the effect of artifacts on segmentation performance. The motion was assessed using 5-level visual grading as described in Wong (2016). We separated the samples into low level of motion determined by grades 1–3, and high level of motion with grades 4 and 5, and compared the performance of all methods. As stated above, 12 out of 20 pQCT scans at 66% tibia contain high to severe motion artifacts (grades 4 and 5).

The next step is to explore the differences in segmentation performance between the two groups. Table 10 summarizes the segmentation performance measured by DSC, true positive rate (TPR) and precision (PR) for each tissue type and each method. This table also contains the relative differences of the performance measures. To estimate the statistical significance of the differences in segmentation performance, we applied nonparametric Wilcoxon rank sum tests between the two groups and we report the *p*-values.

**TABLE 10:** Effect of motion artifacts on segmentation performance.

Dice similarity coefficient (DSC)																
TISSUE	66%-SAT				66%-MUSCLE				66%-CB				66%-TB			
MOTION	Low	High	Diff%	*p*	Low	High	Diff%	*p*	Low	High	Diff%	*p*	Low	High	Diff%	*p*
STPL-FFD	0.799	0.703	−11.9%	0.208	0.919	0.896	−2.5%	0.143	0.691	0.660	−4.5%	0.849	0.785	0.843	7.4%	0.173
STPL-SDD	0.866	0.734	−15.2%	**0.046**	0.964	0.928	−3.8%	**0.004**	0.876	0.810	−7.5%	0.059	0.908	0.914	0.7%	0.849
STPL-SyN	0.794	0.711	−10.5%	0.246	0.926	0.908	−2.0%	0.503	0.792	0.786	−0.8%	0.173	0.845	0.914	8.2%	0.453
STPL-SyNO	0.814	0.697	−14.4%	0.173	0.936	0.901	−3.7%	0.095	0.813	0.747	−8.1%	0.143	0.900	0.856	−4.9%	0.775
TIDAQ	0.922	0.678	−26.4%	**0.014**	0.978	0.876	−10.5%	4.7⋅10^–4^	0.956	0.811	−15.2%	2.3⋅10^–4^	0.960	0.837	−12.8%	**0.035**

TRUE POSITIVE RATE (TPR)																
TISSUE	66%-SAT				66%-MUSCLE				66%-CB				66%-TB			
MOTION	Low	High	Diff%	*p*	Low	High	Diff%	*p*	Low	High	Diff%	*p*	Low	High	Diff%	*p*
STPL-FFD	0.914	0.912	−0.3%	0.703	0.882	0.847	−4.0%	0.443	0.695	0.697	0.2%	0.633	0.952	0.895	−6.0%	0.633
STPL-SDD	0.958	0.901	−5.9%	**0.007**	0.950	0.901	−5.1%	0.117	0.819	0.760	−7.2%	0.336	0.966	0.969	0.3%	0.387
STPL-SyN	0.886	0.890	0.5%	0.775	0.894	0.868	−2.9%	0.775	0.822	0.829	0.8%	0.849	0.925	0.944	2.1%	1.000
STPL-SyNO	0.903	0.888	−1.6%	0.703	0.911	0.860	−5.7%	0.246	0.843	0.772	−8.4%	0.387	0.980	0.887	−9.4%	0.143
TIDAQ	0.911	0.820	−10.0%	0.336	0.978	0.817	−16.4%	2.3⋅10^–4^	1.000	0.982	−1.8%	0.185	0.925	0.774	−16.3%	**0.035**

PRECISION (PR)																
TISSUE	66%-SAT				66%-MUSCLE				66%-CB				66%-TB			
MOTION	Low	High	Diff%	*p*	Low	High	Diff%	*p*	Low	High	Diff%	*p*	Low	High	Diff%	*p*
STPL-FFD	0.732	0.617	−15.7%	0.503	0.964	0.957	−0.6%	0.924	0.719	0.645	−10.3%	0.387	0.672	0.826	23.0%	**0.026**
STPL-SDD	0.797	0.651	−18.3%	0.289	0.980	0.960	−2.0%	0.173	0.958	0.875	−8.7%	**0.002**	0.868	0.870	0.3%	0.775
STPL-SyN	0.747	0.631	−15.5%	0.443	0.966	0.961	−0.6%	0.775	0.794	0.759	−4.4%	0.117	0.791	0.890	12.6%	0.443
STPL-SyNO	0.766	0.617	−19.4%	0.246	0.967	0.956	−1.1%	1.000	0.814	0.740	−9.1%	0.117	0.838	0.839	0.2%	0.566
TIDAQ	0.933	0.613	−34.3%	**0.010**	0.979	0.957	−2.2%	0.117	0.915	0.700	−23.5%	2.3⋅10^–4^	1.000	0.992	−0.8%	1.000

Considering SAT, we observe consistent decrease of average DSC and PR with increasing motion artifacts for all methods. We observe that TIDAQ shows the highest decrease in all performance measures. The Wilcoxon tests indicate statistically significant performance differences for TIDAQ in DSC and precision, and for STPL-SDD in DSC and true positive rate.

### 4.5 Tissue composition assessment

**FIGURE 6:**
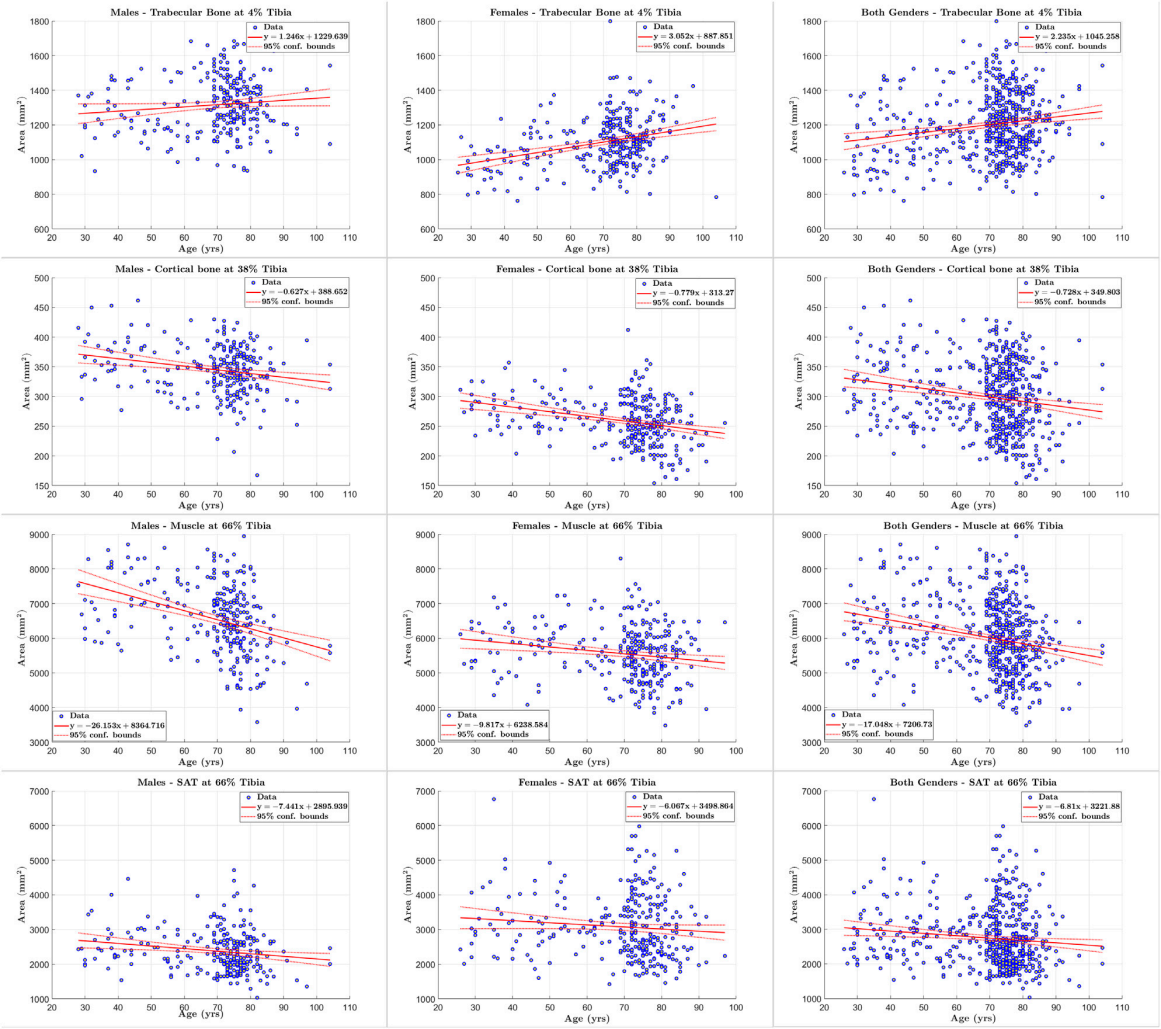
Scatter plots of tissue of interest’s cross sectional area *versus* age for males, females, and both genders at 4%, 38% and 66% tibia.

**FIGURE 7:**
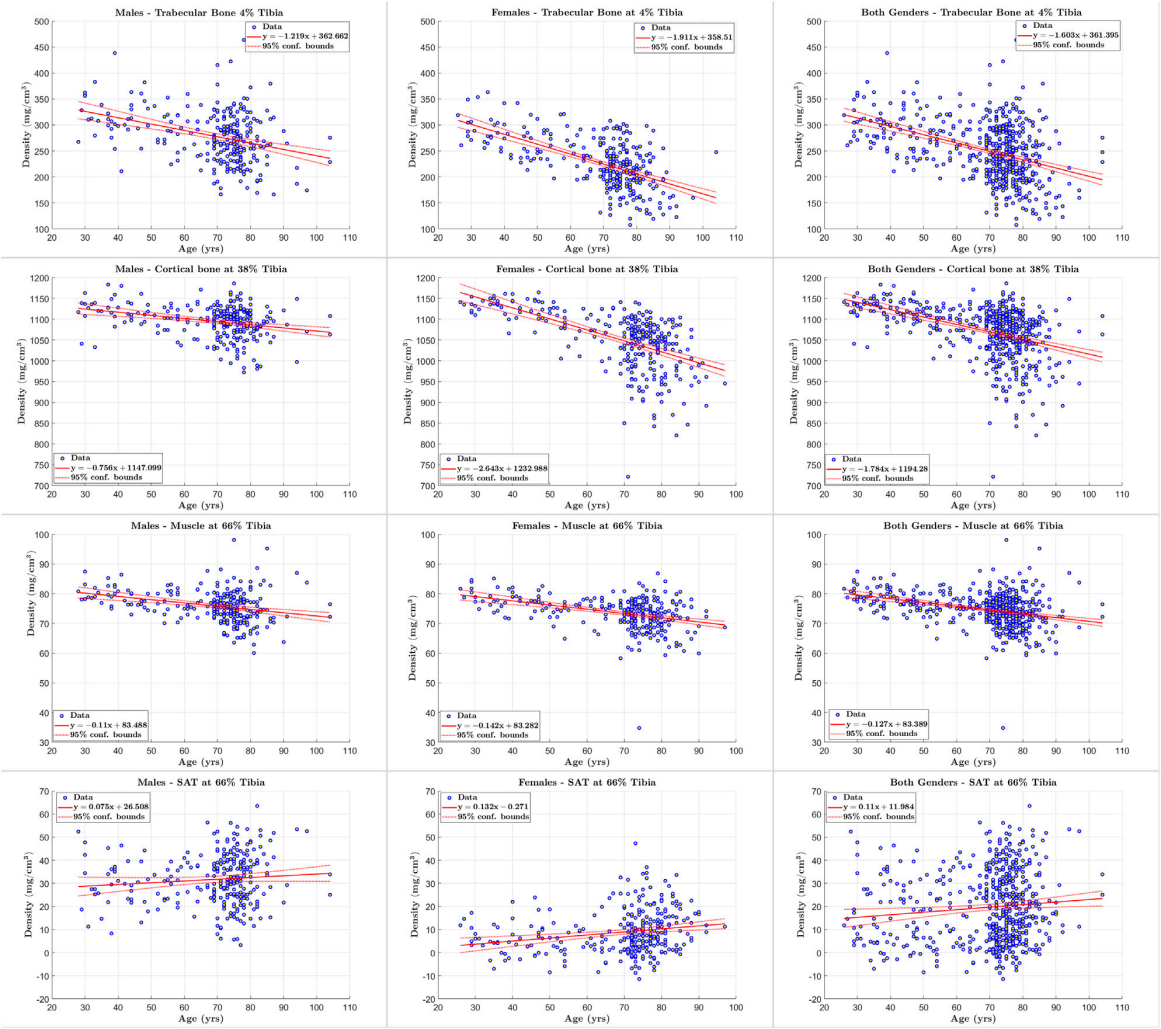
Scatter plots of tissue of interest’s density *versus* age for males, females, and both genders at 4%, 38% and 66% tibia.

We applied our STPL-SDD method to the baseline InCHIANTI dataset that we described in Section 3 and summarized in Table 2. We then analyzed the quantification results of cross-sectional areas and average densities for each tissue to explore changes in its composition as a function of age. The scatter plots and regression results in Figures 6, 7 lead to the following observations. Trabecular bone density decreases with age at similar rates for males and females. The CSA of cortical bone decreases with age at similar rates for males and females. Cortical bone density decreases with age for males and females, and the rate of decrease is higher for females. Muscle CSA decreases with age for males and females, and the rate of decrease is higher for males. Muscle density decreases with age at similar rates for males and females. Our analysis does not reveal significant changes with age for SAT CSA, SAT density, and trabecular bone CSA.

In addition, the statistical results reported in Tables 11–13 show that there is increased correlation of cortical bone density with age, and trabecular bone density with age, especially for females, relative to the other tissues. In addition, there is noticeable correlation between muscle CSA and age for males. The CV-RMSD values show decreased variation mostly for cortical density and muscle density for each gender. We also observe that the gender-conditional analyses produce lower variability than joint analyses of males and females as we expected.

**TABLE 11:** Statistical measures of the relationships between tissue properties and age for males and females.

Measurement	Site	*R* ^2^	CV-RMSD	Slope	Intercept	*p*-value
Trabecular CSA	4	0.031	3.646	2.235	1,045.3	$<10^{-4}$
Trabecular density	4	0.181	4.799	−1.603	361.4	$<10^{-4}$
Cortical CSA	38	0.033	4.661	−0.728	349.8	$<10^{-4}$
Cortical density	38	0.178	1.260	−1.784	1,194.3	$<10^{-4}$
SAT CSA	66	0.013	7.361	−6.810	3,221.9	0.0054
SAT density	66	0.011	18.583	0.110	12	0.0123
Muscle CSA	66	0.058	3.967	−17.048	7,206.7	$<10^{-4}$
Muscle density	66	0.113	1.663	−0.127	83.4	$<10^{-4}$

**TABLE 12:** Statistical measures of the relationships between tissue properties and age for females.

Measurement	Site	*R* ^2^	CV-RMSD	Slope	Intercept	*p*-value
Trabecular CSA	4	0.102	2.159	3.052	887.9	$<10^{-4}$
Trabecular density	4	0.348	3.025	−1.911	358.5	$<10^{-4}$
Cortical CSA	38	0.086	2.528	−0.779	313.3	$<10^{-4}$
Cortical density	38	0.307	0.977	−2.643	1,233	$<10^{-4}$
SAT CSA	66	0.010	5.122	−6.067	3,498.9	0.0870
SAT density	66	0.044	17.826	0.132	−0.3	0.0002
Muscle CSA	66	0.034	2.428	−9.817	6,238.6	0.0013
Muscle density	66	0.150	1.185	−0.142	83.3	$<10^{-4}$

**TABLE 13:** Statistical measures of the relationships between tissue properties and age for males.

Measurement	Site	*R* ^2^	CV-RMSD	Slope	Intercept	*p*-value
Trabecular CSA	4	0.013	1.950	1.246	1,229.6	0.0611
Trabecular density	4	0.127	2.704	−1.219	362.7	$<10^{-4}$
Cortical CSA	38	0.048	1.937	−0.627	388.7	0.0003
Cortical density	38	0.081	0.554	−0.756	1,147.1	$<10^{-4}$
SAT CSA	66	0.032	4.019	−7.441	2,895.9	0.0032
SAT density	66	0.009	5.721	0.075	26.5	0.1141
Muscle CSA	66	0.135	2.362	−26.153	8,364.7	$<10^{-4}$
Muscle density	66	0.087	1.098	−0.110	83.5	$<10^{-4}$

### 4.6 Method implementation and execution time

We developed the programs of the proposed methodologies in C++, *Python* 3.7, and used the ITK library. We implemented the symmetric normalizations, -SyN and -SyNO (originally -SyNOnly) of ANTs on the ANTsPY python library (antspyx version 0.2.5) with default parameters to generate the segmentation results corresponding to these nonlinear registration algorithms. The TIDAQ backend is implemented in C++ and uses ITK, while the user interface is a Java plugin. We executed our experiments on a system with Linux CentOS 7, 2 x Intel(R) Xeon(R) CPU E5-2,690 v4 2.60 GHz, and 128 GB RAM. We computed the execution time of SAIS and MAIS, with respect to the deformable registration algorithms, for all subjects at the different tibia location. We calculated the mean ± standard deviation of the execution time over all subjects in the segmentation set and report the values in Table 14. We observe that the execution time of STAT-SDD is about 27 s for 4% tibia, and about 32 s for 38% and 66% tibia, while the execution time of STPL-SDD is about 102 s for 4%, 118 s for 38%, and 148 s for 66%.

**TABLE 14:** Mean ± standard deviation (in seconds) of the execution times of SAIS and MAIS with respect to nonlinear registration models.

Tibia site	Method	FFD	SDD	SyN	SyNO
4%	SAIS	56.47 ± 3.34	27.01 ± 0.81	5.55 ± 0.30	37.25 ± 2.91
	MAIS	191.45 ± 9.54	101.82 ± 1.72	41.27 ± 0.99	136.48 ± 5.61
38%	SAIS	55.49 ± 7.00	31.15 ± 1.46	5.67 ± 0.33	37.16 ± 1.90
	MAIS	189.31 ± 18.62	117.18 ± 3.97	41.88 ± 1.34	140.61 ± 15.20
66%	SAIS	55.47 ± 3.96	31.48 ± 1.29	4.42 ± 4.42	36.38 ± 1.30
	MAIS	209.66 ± 31.41	147.18 ± 36.05	52.51 ± 13.25	151.13 ± 25.72

## 5 Discussion

### 5.1 Method comparisons, tissue separability and technical characteristics

Tissue delineation is an important, but challenging task in medical image analysis. The accuracy of tissue delineation depends on many factors, including the intrinsic characteristics of the technique, image modality, artifacts or noise, and the number of tissues to be identified in each scan. Peripheral quantitative computed tomography imaging quality is significantly affected by artifacts caused by subject movement. Overall, the proposed multi-atlas image segmentation techniques address the aforementioned factors. Furthermore, the multi-atlas symmetric diffeomorphic demons technique proved to be more robust to reduced image quality than the other methods, followed by symmetric image normalization.

**FIGURE 8:**
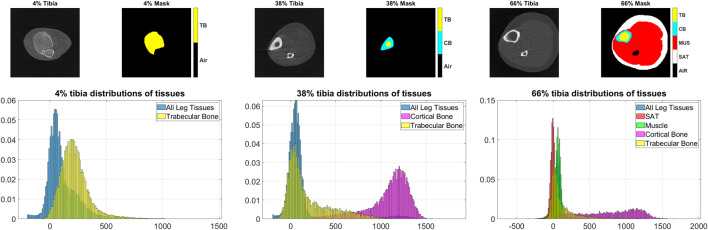
Examples of the pQCT scans, masks and tissue distributions. Top row: image-mask pairs of pQCT scans at 4%, 38%, and 66% tibial length respectively. Bottom: distributions of tissues corresponding to the above tibia sites. On the masks, air, trabecular bone, cortical bone, muscle, and subcutaneous adipose tissue (SAT) are identified by black, yellow, cyan, red, and white colors, respectively.

Visual inspection of tissue densities in the image at multiple tibial sites that are displayed in Figure 8, second row, shows that the distributions of different tissues significantly overlap with one another. At 4% tibia, we observe a clear overlap between the distribution of all leg tissues and the trabecular bone. An optimized thresholding technique based on tissue densities, for example, may not separate the distributions accurately, because of high false positives and false negatives. At 38% tibia, although it appears that there is a valley between the cortical and trabecular bones, yet there is still significant overlap between the distributions of the trabecular bone tissues and all leg tissues. At 66% tibia, we note the extensive overlap among the distributions of SAT, muscle, and trabecular bone. These distributions illustrate the difficulties that would be encountered by segmentation techniques that rely on density alone. Yet, the proposed MAIS technique STPL-SDD, yields high segmentation accuracy (DSC >90%), in almost all tissue delineations at different sites, except for 66%-SAT and 66%-cortical bone.

Specific properties of these methods that improved the tissue delineation accuracy are their 1) symmetric nature, and 2) diffeomorphism. Concerning symmetry, the method in question provides equal treatment to both fixed and moving images. In addition, the interactive force between the two images can produce accurate registration of one part of the image to the other, and *vice versa* (Rogelj and Kovačič, 2006). On the other hand, diffeomorphism affords the algorithms the ability to handle both large and small deformations (Sotiras et al., 2013). It is important to note that large deformations are a result of large strains or rotations, which are caused by subject movement. Thus, symmetric diffeomorphic demons and symmetric normalization are robust to local image artifacts or large image deformations that are difficult to register.

The segmentation results shown in Figure 4, at 4% and 38% tibial length, correspond to subjects corresponding to variable levels of delineation challenges, caused by either the condition of the subject (in the 4% examples) or subject motion (in the 38% examples). At both anatomical sites, subject ‘B’ presents more segmentation challenges than subject ‘A’. Visual inspection of subject ‘B’ at 4% tibia in Figure 4 shows that STPL-FFD and STPL-SyNO could not delineate the trabecular bone accurately when compared to STPL-SDD and STPL-SyN. Similarly, under 38%-tibia scans in Figure 4, we observe that due to the higher presence of streaks in subject ‘B’ than in subject ‘A’, STPL-FFD and STPL-SyNO produced lower tissue delineation accuracy than STPL-SDD and STPL-SyN. This observation provides insight into the improved delineation accuracy produced by symmetric diffeomorphic demons and symmetric normalization.

### 5.2 Effects of subject motion on tissue identification at 66% tibia

The effect of motion artifacts is less pronounced on muscle identification in terms of the relative differences. We observe statistically significant differences in DSC for STPL-SDD and TIDAQ. TIDAQ also showed statistically significant decrease of muscle true positive rate.

In the cortical bone, most performance changes are not statistically significant. TIDAQ produced *p*-values smaller than 0.05 for DSC and precision, and STPL-SDD for precision only. In the trabecular bone, STPL-FFD produced *p*-values smaller than 0.05 for precision, and TIDAQ for DSC and true positive rate. DSC and precision values of the trabecular bone produced by STPL-FFD and STPL-SyN increase from low to high motion group, because of segmentation errors in subjects of the low motion group.

We observe that TIDAQ is significantly affected by motion artifacts, as average DSC clearly decreases from the low to high subject motion group. MAIS techniques and especially STPL-SyN are more resilient to subject motion. STPL-SDD still produces the highest DSC and precision values in the high motion group overall.

### 5.3 Tissue composition assessment

The results in Figures 6, 7 and Tables 11–13 are consistent with findings of clinical studies of aging that used semi-manual quantification workflows (Makrogiannis et al., 2018; Ferrucci et al., 2000). These results indicate that our automated methodology can help to increase the throughput of sophisticated cross-sectional and longitudinal analyses of tissue properties. We also note that the proposed methodology enables the analysis of both hard and soft tissues in pQCT. This is a desirable and innovative feature as pQCT has been mostly restricted to quantification of bone in the past (Gabel et al., 2018; Wong and Manske, 2020). Our methodology opens the door for efficient exploration of muscle properties in the lower leg using pQCT. On the other hand, a greater number of reference segmentation masks may be needed to improve the statistical power of performance evaluations.

## 6 Conclusion

We introduced multi-atlas segmentation methods for soft and hard tissue segmentation in the lower leg using pQCT data. Our results indicate that the MAIS technique, STPL-SDD, produced more accurate tissue delineation as measured by DSC than all compared methods. STPL-SyN is largely resilient to subject motion artifacts and noise. The results of our experiments indicate that our methodology can analyze data with degradations caused by subject motion that conventional methods cannot analyze. Future directions of this work include extending this framework to 3D imaging data, and using the segmentation and quantification results for disease prognosis and diagnosis.

## Data Availability

The raw data supporting the conclusions of this article will be made available by the authors, without undue reservation.
